# Circular RNA Pvt1 oncogene (CircPVT1) promotes the progression of papillary thyroid carcinoma by activating the Wnt/β-catenin signaling pathway and modulating the ratio of microRNA-195 (miR-195) to vascular endothelial growth factor A (VEGFA) expression

**DOI:** 10.1080/21655979.2021.2008639

**Published:** 2021-12-19

**Authors:** Linwen Zeng, Shaofeng Yuan, Pengfei Zhou, Jianming Gong, Xiangdong Kong, Ming Wu

**Affiliations:** Department of Surgery, Tinglin Hospital of Jinshan District, Shanghai, China

**Keywords:** Papillary thyroid carcinoma, *circPVT1*, *miR-195*, *VEGFA*, malignant progression

## Abstract

Circular RNAs (circRNAs) have been reported to be involved in the progression of papillary thyroid carcinoma (PTC). However, the role of circular RNA Pvt1 oncogene (*circPVT1)* in PTC has rarely been reported. In this study, we aimed to investigate the function and mechanism of *circPVT1* in PTC. The expression level of *circPVT1, miR-195* and *VEGFA* were determined by reverse transcription‑quantitative PCR (RT‑qPCR). Fisher’s exact test was used to analyze the correlation between *circPVT1* expression and PTC clinical features. Cell Counting Kit-8 (CCK-8) and 5-Ethynyl-2ʹ-deoxyuridine (EdU) staining assay and transwell assay were conducted to evaluate the cell proliferation, migration and invasion ability. Dual-luciferase reporter and Western blot assay were conducted for evaluating the correlation between *miR-195* and *circPVT1* or *VEGFA*. The results of RT-PCR showed that the expression level of *circPVT1* was significantly upregulated in PTC tissues and cell lines. After downregulating *circPVT1* expression in PTC cells, the abilities of cell proliferation, migration, and invasion were obviously suppressed, and the Wnt/β-catenin signaling pathway was also repressed. Besides, *miR-195* could both bind to *PVT1* and *VEGFA*, while *PVT1* could promote the expression of *VEGFA* by binding to *miR-195*. Downregulation of *VEGFA* expression in PTC cells revealed weakened cell proliferation, migration, and invasion capacities, and restrained Wnt/β-catenin signaling pathway. Therefore, we demonstrated that *circPVT1* could promote *VEGFA* expression by sponging *miR-195. CircPVT1* could serve as a molecule sponge for *miR-195* and mediate the ceRNA network to promote the expression of *VEGFA*, thus contributed to the malignant progression of PTC.

## Introduction

Thyroid cancer (TC) is one of most prevalent endocrine malignancy worldwide, and its incidence has doubled globally in the last 30 years [1–3], TC is one of the reasons for the high incidence of malignant tumors in women, as it is the fifth most diagnosed malignant tumor in this group [4,5]. Papillary thyroid cancer (PTC) is the most common histotype of TC, accounting for approximately 85% of all TC [6]. Although the prognosis of PTC is generally good, a high quality of life still cannot be guaranteed. This is because some PTC patients show early epidural infiltration, lymphatic metastasis, and even distant metastasis or other high-risk complications [7]. More than 30% of PTC patients develop into lymph node metastasis [8], while 2.6% to 3.7% of patients suffer from distant metastasis [5,9]. The occurrence of distant metastasis and invasion of PTC indicates a poor prognosis, and often the disease cannot be effectively controlled [10]. Consequently, investigating the mechanism of the malignant progression of PTC has vital theoretical significance and potential clinical value.

Circular RNAs (circRNAs) were discovered approximately 40 years ago [11], but have not attracted widespread attention until recently. As circRNAs are ncRNAs, they were initially thought to be byproducts of pre-mRNA splicing errors [12]. Compared with other types of noncoding RNAs (ncRNAs), circRNAs have neither 5ʹ to 3ʹ polarity nor a polyA tail, because they form covalently closed loops. These circRNAs are more conserved and stable than linear ncRNAs [13,14]. In addition, some studies have found that there are four basic types of circRNAs [15]: exon circRNAs, intronic circRNAs, exon-intron circRNAs, and intergenic circRNAs. With the rapid development of next-generation sequencing technology, many circRNAs have been identified in mammalian transcriptomes and various cancers [16–18]. Because circRNA is not easily degraded by exonuclease RNase R, most of them have a long half-life and are widely present in tissues and peripheral blood, and they are highly conserved and have spatial tissue and disease specificity [19,20]. These characteristics endow it with a potential function, such as acting as a miRNA molecular sponge, regulating gene expression and participating in protein translation, and may also be a potential target for disease intervention [21,22]. In addition, circRNA in peripheral blood has stable biological properties and is expected to become a molecular marker for disease diagnosis and prognosis [16,23]. In human cancers, substantial researches have demonstrated that circRNAs may play an essential role in cancer growth, metastasis, and malignant progression. For example, Li et al. find that circRNA *hsa_circ_0014130* can act as a *miR-132-3p* sponge for playing oncogenic roles in bladder cancer by upregulating potassium inwardly rectifying channel subfamily J member 12 (*KCNJ12)* expression [24]. Zhang et al demonstrate that *hsa-circ_0058106* can induce epithelial-mesenchymal transition (EMT) and metastasis in laryngeal cancer via sponging *miR-153* and inducing twist family bHLH transcription factor 1 (*TWIST1)* nuclear translocation [25]. Cai et al show that circ-NOL10 regulated by metadherin (*MTDH*)/CASC3 exon junction complex subunit (*CASC3*) can suppress breast cancer progression and metastasis via bind to multiple miRNAs and programmed cell death 4 (PDCD4) [26]. These studies identify that aberrant circRNAs expression may be encouraging diagnosis biomarkers and therapeutic target for PTC.

The circRNA *PVT1* (*circPVT1*) is a recently identified circRNA situated in the known cancer susceptibility region chr8: 128902834–128903244 [27,28]. It originates from the third exon of the *PVT1* and has been identified as an oncogene [27]. Previous studies have shown that *circPVT1* may play a vital role in the ininiation and progression of several tumors, including laryngeal cancer [29], non-small cell lung cancer [30], gastric cancer [31], clear cell renal cell carcinoma [32], ovarian cancer [33], cervical cancer [34] and breast cancer [35]. However, the expression pattern and biological function of *circPVT1* in PTC remain unknown. In the current study, we hypothesized that *circPVT1* might assume an essential role in the progression of PTC. The aim of this study was to reveal the biological function and potential molecular mechanism of *circPVT1* regulating the progression of PTC. We firstly measured *circPVT1* expression level in the PTC tissues and cell lines. Meanwhile, we detected the functions of *circPVT1* on the cell growth, migration, and invasiveness of PTC. Finally, we revealed the underlying mechanism that circVRK1 sponged miR-195 and accelerated the VEGFA expression, so as to regulate the Wnt/β-catenin signaling pathway.

## Materials and methods sample Collection

Fifty pairs of PTC tumor samples and matching contralateral normal samples were collected from patients with PTC in our hospital between October 2015 and March 2017. The samples were frozen in liquid nitrogen and stored at −80°C until use. We verified the cancer status of all the tissue samples by pathological examination. The Ethics Committee of the Tinglin Hospital of Jinshan District approved the study (2015–023) and informed consent was obtained from patients before surgical operation.

### Cell culture

We purchased PTC cell lines (K1, IHH-4, BCPAP, and TPC-1) and human thyroid follicular epithelial cells (Nthy-ori 3–1) from the Hematology Institute (Beijing, China) of the Chinese Academy of Chinese Medical Sciences. The K1, IHH-4 and Nthy-ori 3–1 cells were cultured in Roswell Park Memorial Institute (RPMI)-1640 medium (Invitrogen, Grand Island, NY, USA), while TPC-1 and BCPAP cells were cultured in Dulbecco’s Modified Eagle Medium (DMEM). Additional MEM Non-Essential Amino Acids Solution (NEAA 100X) (Invitrogen, 11140050) was added to the BCPAP cell line. All medium was containing 10% heat-inactivated fetal bovine serum (FBS; Invitrogen), penicillin (100 U/mL)/streptomycin (100 μg/mL). The cells were incubated in a humidified incubator at 37°C and 5% CO_2_.

### Cell transfection

We purchased the *PVT1* small interfering RNA (siRNA), VEGFA siRNA, miR-195 inhibitor, and their corresponding negative controls from GenePharma (Shanghai, China). Next, we mixed the cells in a 6-well plate (5 × 10^5^ cells per well). Following the manufacturer’s instructions, after the cell density reached 80%, the transfection reagent was mixed with Lipofectamine 2000 (Invitrogen) and added to the cells [36]. The mixture was incubated at room temperature for 30 min and subsequently transferred to a Petri dish. After transfection for 48 h using Lipofectamine 2000, the transfected cells were collected to assess the transfection efficiency by qRT-PCR. The sequences of primers for transfection were listed below: circPVT1 siRNA: 5ʹ-GCAAAUGAAAGCUACCAAUTT-3ʹ; scramble siRNA: 5ʹ-UUCUCCGAACGUGUCACGUTT-3ʹ; VEGFA siRNA: 5ʹ-CUGGAAUUUGAUAUUCAUUGA-3ʹ; scramble siRNA: 5ʹ-UUCUCCGAACGUGUCAC GUTT-3ʹ; miR‑195 mimics sense, 5ʹ‑UAGCAGCA CAGAAAUAUUGGC‑3ʹ; miR‑195 mimics antisense, 5ʹ‑CAAUAUUUCUGUGCUGCUAUU‑3ʹ; mimics negative control (miR‑NC) sense, 5ʹ‑UUC UCCGAACGUGUCACGUTT‑3ʹ; miR‑NC antisense, 5ʹ‑ACGUGACACGUUCGGAGAATT‑3ʹ.

### RNA extraction and reverse transcription‑quantitative PCR (RT‑qPCR)

After transfection for 24–48 h, purified total RNA was extracted from tissues and cells using TRIzol (Invitrogen). cDNA was obtained by reverse transcription in a 20 μL reaction system using the PrimeScript RT reagent Kit (TAKARA, Code No. RR037A) according to the manufacturer’s instructions. Real-time quantitative reverse transcription polymerase-chain reaction (RT-PCR) analysis was performed to determine transfection efficiency using the following conditions: 92°C for 10 min, followed by 40 cycles of 92°C for 10 s and then 60°C for 1 min [37]. GAPDH and U6 were used as the internal reference, respectively. The primer sequences used were as follows: PVT1 (F: 5ʹ-GGTTCCACCAGCGTTATTC-3ʹ; R: 5ʹ-CAACTT CCTTTGGGTCTCC-3ʹ); miR-195 (F: 5ʹ-ACGA TGCCCACGACCAAGCC-3ʹ; R: 5ʹ-AGCACCATC GTCCGCAGGCA-3ʹ); VEGFA (F: 5ʹ-AACTTT CTGCTGTCTTGGGT-3ʹ; R: 5ʹ-TCTCGATTGGA TGGCAGTA-3ʹ); Wnt3a (F: 5ʹ-TCTACGACGT GCACACCTG-3ʹ; R: 5ʹ-CCCTGCCTTCAGGTA GGAGT-3ʹ); β-catenin (F: 5ʹ-CAGCAGCAATT TGTGGAGGG-3ʹ; R: 5ʹ-GCAGCTGCACAAACA ATGGA-3ʹ); GAPDH (F: 5ʹ-GGAATCCACTGG CGTCTTCA-3ʹ; R: 5ʹ-GGTTCACGCCCATCAC AAAC-3ʹ); U6 (F: 5ʹ-CTCGCTTCGGCAGCACA-3ʹ; R: 5ʹ-AACGCTTCACGAATTTGCGT-3ʹ).

### Cell proliferation activity assay

We used Cell Counting Kit-8 (CCK-8, Dojindo, Kumamoto, Japan) to detect cell growth ability and the method referred to the previous study [38]. Cells in the medium were digested with 0.25% trypsin and the cell suspensions were subsequently harvested and seeded into 96-well plates, with 6 wells per group. Each well contained at least 2 × 10^3^ cells and 200 μL of the medium. The cells were incubated overnight for adherent growth and the cell supernatant was washed with phosphate-buffered saline. A mixture containing 90 μL of pure 1640 medium and 10 μL of CCK-8 solution (Beyotime Biotechnology, Shanghai, China) was added to each well. After 2 h of incubation, the absorbance of each well was measured at 450 nm using a microplate reader.

### Transwell experiment

The cell invasiveness was examined with the 8-mm transwell (Millipore, Billerica, MA, USA)

with (invasion assays) or without (migration assays) the coating of Matrigel. According to previous study [39], the cells were seeded into serum-free medium and added to the upper chamber (1 × 10^4^ cells per chamber) coated with 200 mg/mL of Matrigel. A medium containing 10% FBS was added to the lower chamber as a chemical attractant. After 24 h of incubation, the cells in the upper chamber were removed by wiping with a cotton swab. We then fixed the cells invading the lower surface of the filter in 70% ethanol for 30 min, and stained them for an additional 10 min with 0.2% crystal violet. The invading cells were photographed and counted across five random fields of view in each cell under an optical microscope at 200× magnification.

### 5-ethynyl-2ʹ-deoxyuridine (EdU) assay

The EdU assay was conducted according to previous study [40]. The cells were pre-inoculated in a 24-well plate at a density of 5 × 10^3^ cells per well. The plated cells were then incubated in 4% methanol for 30 min, followed by permeabilization in 0.5% TritonX-100 for 10 min, and a 30-min reaction in 400 μL of 1× ApollorR. Afterward, the cells were stained with 4ʹ,6-diamidino-2-phenylindole for another 30 min. The EdU-stained cells were counted under a fluorescence microscope (CKX41-F32FL, Olympus, Tokyo, Japan) to calculate the percentage of EdU-positive cells.

### Western blot analysis

The experimental method of Western blot referred to the previous literature [41]. Total protein was extracted from the transfected cells and the equal quantity proteins (30 μg) were separated using a 10% polyacrylamide gel and transferred to a 0.22 μm polyvinylidene fluoride membrane. The membranes were then incubated in blocking buffer (5% skim milk) for 2 h, and then incubated with primary antibodies at 4°C overnight. After incubation with the corresponding secondary antibodies for 2 h at room temperature, the protein bars were visualized by chemiluminescence using the ECL reagent (Pierce, Rockford, IL, USA). The antibodies used were as follows: Wnt3a (ab219412, 1/1000, abcam), β-catenin (ab32572, 1/5000, abcam), β-actin (ab8226, 1/1000, abcam), VEGFA (ab52917, 1/10000, abcam) and the anti-rabbit and anti-mouse peroxidase-conjugated secondary antibodies (Santa Cruz Biotechnology).

### Dual luciferase reporter experiment

We used bioinformatics websites (starbase, https://starbase.sysu.edu.cn/) to predict the potential targets of *miR-195, PVT1*, and *VEGFA* [42]. A PVT1 3ʹ-UTR or a mutant PVT1 3ʹ-UTR with a predicted target site was inserted into a pGL3-promoter vector. Cells in the logarithmic growth phase were seeded into a 96-well plate at a density of 1.5 × 10^4^ cells per well and cultured in an incubator for 24 h. After co-transfecting the cells for 48 h with a final concentration of 50 nmol/L of miRNA mimics or a non-target control, and 100 ng of the dual-reporter gene vector with a target gene 3ʹ UTR or a mutant vector 3ʹ UTR, the medium was aspirated and 35 μL fresh medium added per well. Next, 35 μL of luciferase (Promega, Madison, WI, USA) substrate was added to each well. After shaking for 10 min, the fluorescence intensity was measured. The experiment was repeated 3 times.

### Statistical analysis

Data analysis was performed with the SPSS software (SPSS ver. 17.0, SPSS Inc., Chicago, IL, USA), and all the figures were processed with the GraphPad Prism software (Version X; La Jolla, CA, USA). Comparisons between two groups were analyzed using Student’s t-test. One-way analysis of variance (ANOVA) followed by Tukey’s test was utilized to analyze the comparisons among more than two groups. Fisher’s exact test was used to analyze the correlation between circPVT1 expression and PTC clinical features. The Kaplan–Meier method was applied to calculate the survival rate of patients. The results were displayed as mean ± standard deviation (SD). Statistical significance was set at *p* < 0.05.

## Results

This study aimed to reveal the expression pattern, biological function and potential molecular mechanism of *circPVT1* in PTC. We hypothesized that *circPVT1* might act as a significant role in the progression of PTC. In basis of our results of in vitro experiments, we demonstrated that *circPVT1* was obviously up-regulated in osteosarcoma tissues and cell lines, and *circPVT1* could sponge *miR-195* to up-regulate *VEGFA* expression to promote PTC progression.

### CircPVT1 was highly expressed in cells from PTC tissue

**Figure 1.**
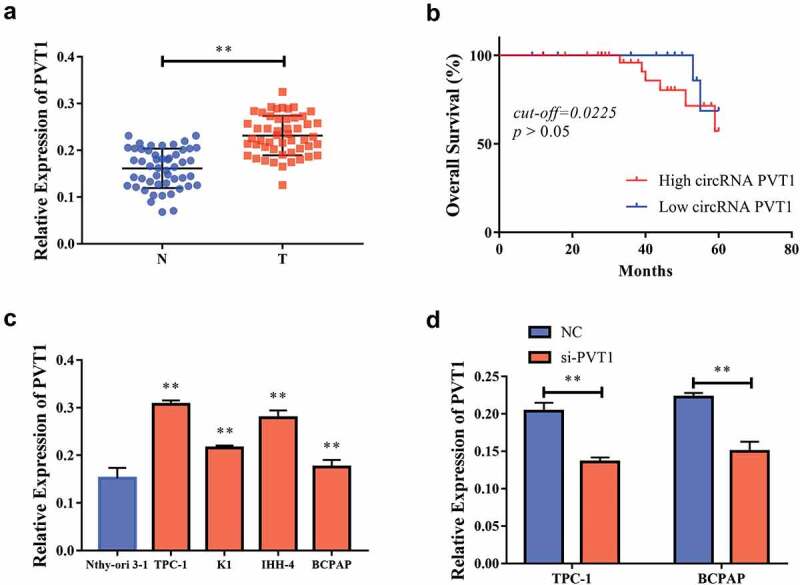
CircPVT1 had high expression in PTC tissue. a. Compared to cancer-side tissue, the level of PVT1 expression in thyroid papillary carcinoma tissue was significantly increased; b. Statistically analyzed the expression level of PVT1 had no significant correlation with PTC patients; c. Compared to human thyroid follicle epithelial cells (Nthy-ori 3–1), pvT1 was significantly high in thyroid papillary cell lines (K1, IHH-4, BCPAP, TCP1); d. PvT1 expression was significantly reduced after TPC-1 and BCPAP cells transfected si-PVT1. (** P < 0.01, the expression level of PVT1 was normalized to U6)

Through RT-PCR, we found that the level of *PVT1* expression in PTC tissue was significantly higher than that in non-cancerous tissue Figure 1(a). Meanwhile, according to the median of circPVT1 level, we divided 50 cases into two groups, namely, Circ PVT1 High group (n = 25) and circPVT1 Low group (n = 25). The Kaplan–Meier curve indicated that the expression level of circPVT1 had no significant correlation with the overall survival rate of PTC patients (cut-off = 0.225, *p*> 0.05) Figure 1(b). We also analyzed the correlation between the expression levels of *PVT1* and special clinical pathological parameters. As shown in Table 1, the expression level of *PVT1* was significantly related to tumor size, TNM stage, and lymph node metastasis in patients with PTC. Compared to Nthy-ori 3–1 cells, the *PVT1* expression level in PTC cell lines (K1, IHH-4, BCPAP, and TCP1) increased significantly Figure 1(c). We selected TPC-1 and BCPAP cells for further experiments. First of all, we transfected *si-PVT1* into BCPAP cells and assessed the transfection efficiency. We observed a significant decrease in the *PVT1* expression levels in the BCPAP cells due to this transfection Figure 1(d).

**Table 1.** Relationship between circPVT1 expression and the clinical pathological characteristics of PTC patients (n = 50)

Clinic pathological features		NO. of cases	CircPVT1 (n, %)		*p* – value
			Low	High	
Gender	Male	29	14	15	*P* > 0.05
	Female	21	11	10	
Age	≤45	34	15	19	*P* > 0.05
	> 45	16	7	9	
Extra thyroidal extension	Negative	17	7	10	*P* > 0.05
	Positive	33	14	19	
Tumor size	≤1	36	25	11	*P* < 0.05
	>1	14	4	10	
TNM stage	I/II	28	20	8	*P* < 0.05
	III/Ⅳ	22	7	15	
Lymph node metastasis	≤45	23	16	7	*P* < 0.05
	>45	27	9	18	
Nodular Goiter	Negative	31	15	16	*P* > 0.05
	Positive	19	9	10

### Downregulation of PVT1 could inhibit the proliferation, migration, and invasion of PTC cells

**Figure 2.**
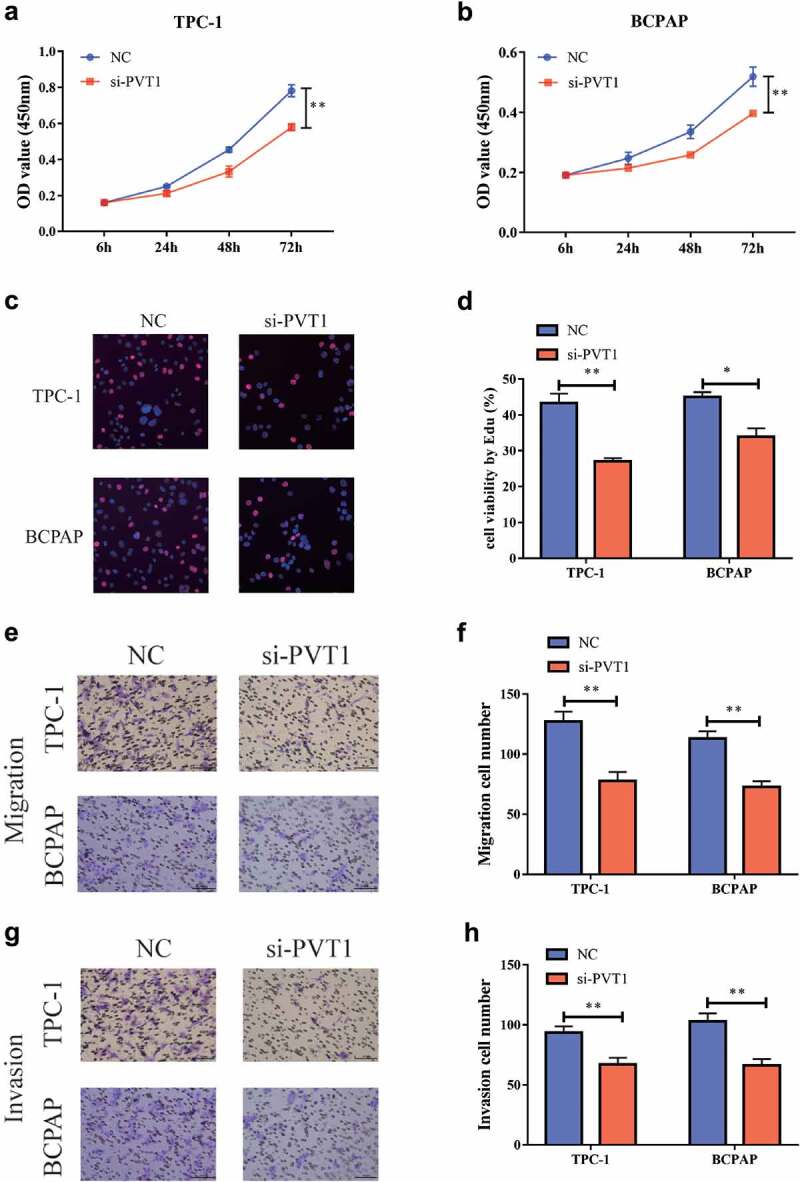
Down-regulation of circPVT1 could restrain the multiplication, migration as well as aggression of thyroid papillary carcinoma cells. a. CCK-8 test showed that when reducing PVT1 expression in TPC-1 cells, cell proliferation was reduced; b. CCK-8 experiments showed that when PVT1 expression was reduced in BCPAP cells, cell multiplication was reduced; c, d. Flat-panel cloning experiments showed that in TPC-1 as well as BCPAP cells, when reducing PVT1 expression, cell multiplication ability decreased; e, f. Transwell experiments showed that the reduction of PVT1 expression in TPC-1 and BCPAP cells, and the ability to migrate cells decreased; g, h. Transwell experiments showed that the cell attack ability decreased when reducing the expression of PVT1 in TPC-1 as well as BCPAP cells. (** P < 0.01)

The effect of the *PVT1* expression level on the PTC cells was detected using a CCK-8 test. After the reduction of *PVT1* expression in TPC-1 and BCPAP cells, the cell proliferation capacity was significantly inhibited Figure 2(a,b). Similar results were observed in the EdU experiment Figure 2(c,d). The transwell assay showed that after the downregulation of *PVT1* expression, cell migration Figure 2(e,f), and invasiveness were also significantly reduced Figure 2(g,h). Based on the above observations, we concluded that reducing *PVT1* expression can significantly inhibit the proliferation, migration, and invasion of PTC cells.

### Downregulation of PVT1 could inhibit the Wnt/β-catenin signaling pathway

**Figure 3.**
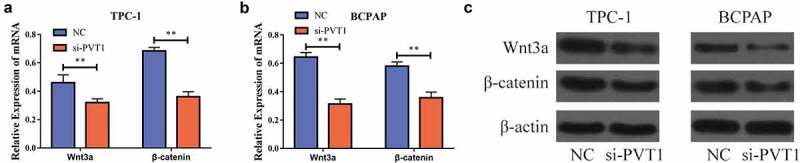
circPVT1 could activate the Wnt/β-catenin signal pathway. a. Down-regulation of PVT1 expression in TPC-1 cells inhibits mRNA expression of Wnt/β-catenin signal pathway-related genes; b. Lower PVT1 expression in BCPAP cells inhibits the mRNA expression of Wnt/β-catenin signal pathway-related genes; c. Western blot showed that the level of gene proteins related to the Wnt/β-catenin signal pathway decreased when PVT1 expression was lowered in TPC-1 as well as BCPAP cells. (** P < 0.01)

To further explore the specific role of *PVT1* in PTC development, we lowered the expression of *PVT1* in the PTC cells and detected the expression of genes related to the Wnt/β-catenin signaling pathway using RT-PCR. The results showed that following the inhibition of *PVT1* expression, the mRNA expression of these genes was significantly decreased Figure 3(a,b). Furthermore, the level of proteins related to Wnt/β-catenin signaling pathway-related genes was also significantly reduced Figure 3(c). Thus, we found that following the inhibition of *PVT1* expression, the expression of the Wnt/β-catenin signaling pathway was also significantly inhibited. This indicated that *PVT1* could play a role in activating the Wnt/β-catenin signaling pathway.

### PVT1 could target miR-195 and regulate its expression

**Figure4:**
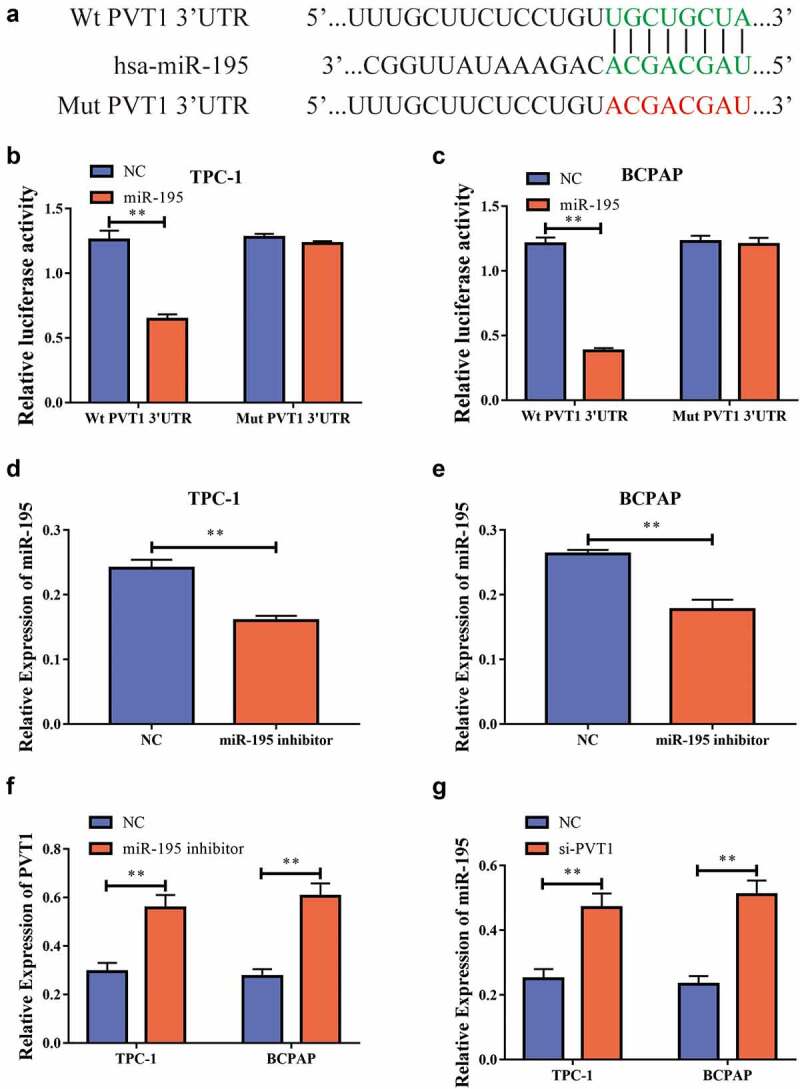
circPVT1 could target miR-195. a. Bioinformatics predicts potential binding sites of PVT1 and miR-195; b. Dual luciferase reporting genetic experiment of TPC-1 cells shows that PVT1 can target miR-195; c. Dual luciferase reporting genetic experiment in BCPAP cells shows that PVT1 can target miR-195; d. After transfecting the miR-195 inhibitor into the TPC-1 cell, the expression level of miR-195 decreased; e. BCPAP cells have reduced the level of miR-195 expression after transiting miR-195 pontoon; f. PVT1 expression levels rises after down-regulation of miR-195 expression in TPC-1 as well as BCPAP cells; g. MiR-195 expression levels rises after the expression of PVT1 was reduced in TPC-1 and BCPAP cells. (** P < 0.01, the expression level of PVT1 was normalized to U6)

We used bioinformatics to predict potential binding targets for *circPVT1* and *miR-195* Figure 4(a), and then constructed wild-type and mutant sequences of *PVT1* (PVT1-WT and PVT1-MUT, respectively). Using dual-luciferase reporter gene experiments, we discovered that after the transfection of *miR-195* mimics into the TPC-1 and BCPAP cells, the luciferase level in the PVT1-WT 3ʹUTR group was reduced, while there was no significant difference in the luciferase level in the PVT1-MUT 3ʹUTR group Figure 4(b,c). This result implied that *PVT1* could target miR-195. To further study the regulatory relationship between the two, we transfected the cells with an miR-195 inhibitor. Using RT-PCR, we found that inhibition resulted in significantly decreased *miR-195* expression levels after transfection Figure 4(d,e), and significantly increased *PVT1* expression levels Figure 4(f). In addition, we found that *miR-195* expression was significantly increased after *PVT1* expression was downregulated Figure 4(d). Thus, we found that *PVT1* could target *miR-195* and inhibit its expression.

### PVT1 could regulate VEGFA expression through miR-195

**Figure5:**
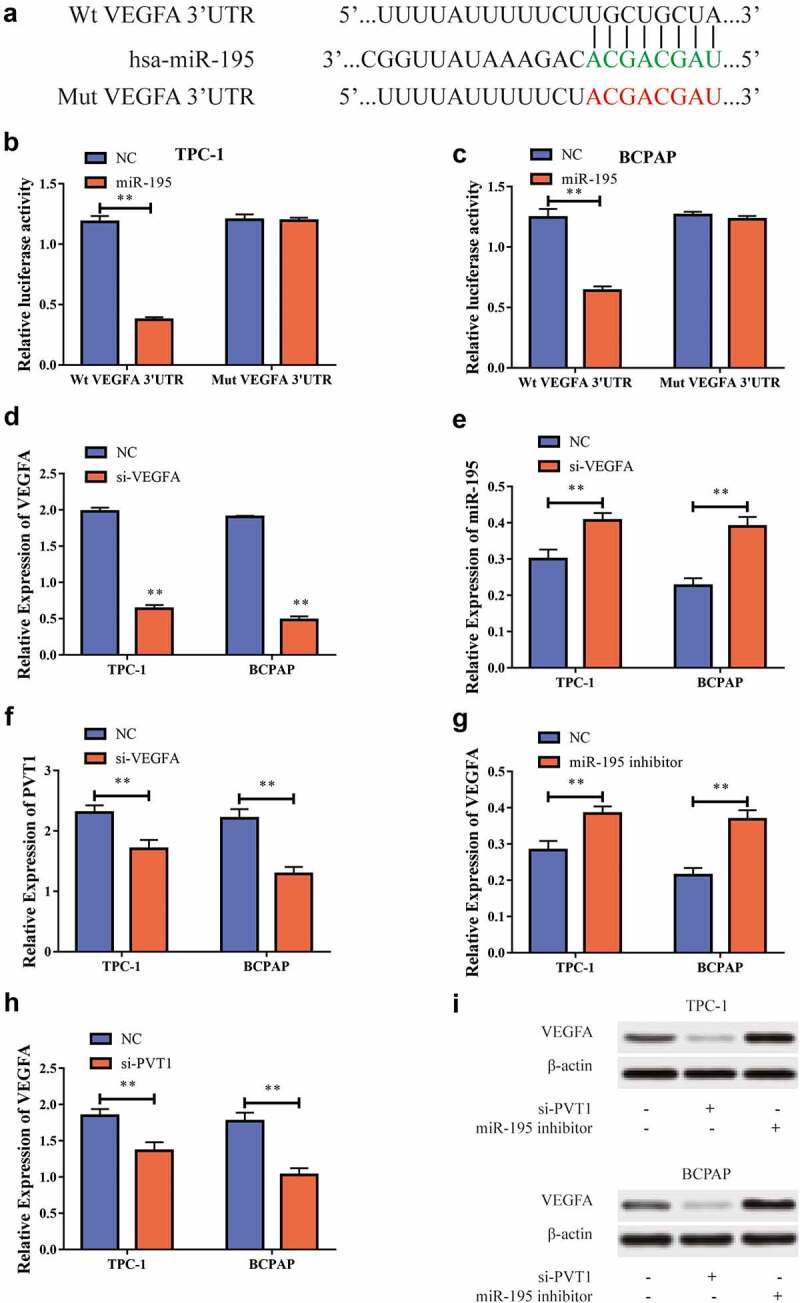
miR-195 could target VEGFA. a. Bioinformatics predicts the potential binding sites between miR-195 and VEGFA; b. Dual luciferase reporting gene experiment in TPC-1 cells shows that miR-195 can target VEGFA; c. Dual luciferase reporting gene experiment in BCPAP cells shows that miR-195 can target VEGFA; d. After transfecting si-VEGFA into TPC-1 as well as BCPAP cells, VEGFA expression was reduced; e. MiR-195 expression levels rises after down-regulating expression of VEGFA in TPC-1 and BCPAP cells; f. PVT1 expression level declines after VEGFA expression is lowered in TPC-1 and BCPAP cells; g. VEGFA expression levels rose after downward expression in TPC-1 and BCPAP cells; h. The expression of VEGFA is reduced after PVT1 expression is reduced in TPC-1 and BCPAP cells; i. Western blot shows that when lowering PVT1 expression in TPC-1 as well as BCPAP cells, VEGFA protein levels decrease; when miR-195 is downgraded, VEGFA protein levels increase. (** P < 0.01, the expression level of VEGFA was normalized to GAPDH)

We also used bioinformatics to predict potential binding targets for *miR-195* and *VEGFA* Figure 5(a) and then constructed *VEGFA* wild-type and mutant sequences (VEGFA-WT and VEGFA-MUT, respectively). Using a dual luciferase reporter gene experiment we found that, after transfection of *miR-195* mimics into TPC-1 and BCPAP cells, the luciferase levels in the VEGFA-WT 3ʹUTR group decreased, while those in the VEGFA-MUT 3ʹUTR showed no significant difference Figure 5(b,c). This suggested that *miR-195* can target *VEGFA*. To further study the regulatory relationship between the two, we transfected cells with *si-VEGFA* to inhibit its expression. RT-PCR showed that following transfection, the *VEGFA* expression level in the cells was significantly reduced Figure 5(d), while the *miR-195* expression level significantly increased Figure 5(e), and the *PVT1* expression level decreased significantly Figure 5(f). To further explore this regulatory behavior, we used RT-PCR to show that *VEGFA* expression levels increased after downregulation of *miR-195* expression Figure 5(g), and also that *VEGFA* expression was significantly suppressed after *PVT1* expression was suppressed Figure 5(h). Western blot analysis also showed the same results at the protein level Figure 5(i). Based on the above results, we concluded that *PVT1* could regulate the expression of *VEGFA* through targeted binding with miR-195 in the PTC cells.

### Lowering VEGFA restrained the growth, migration, and invasion of PTC cells

**Figure 6.**
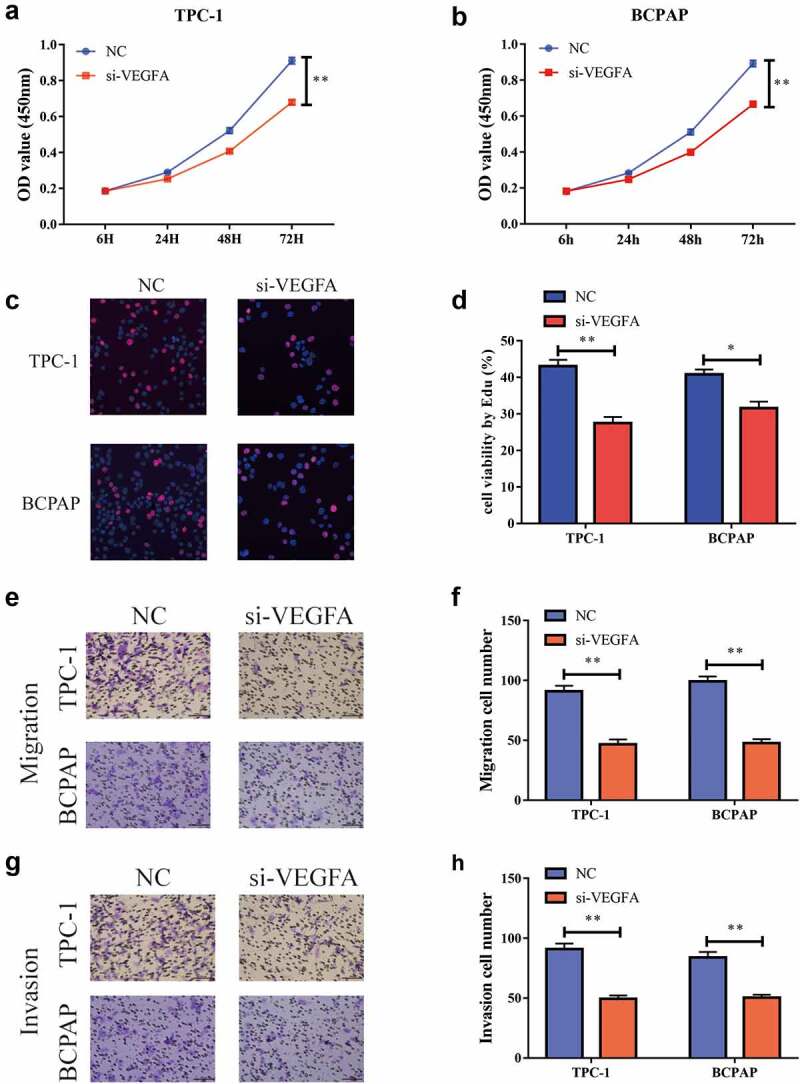
Down-regulation of VEGFA could restrain the multiplication, migration as well as aggression of thyroid papillary carcinoma cells. a. CCK-8 test shows that cell proliferation is reduced when reducing VEGFA expression in TPC-1 cells,; b. CCK-8 test shows that VEGFA expression is reduced in BCPAP cells, cell multiplication is reduced; c, d. Flat-panel cloning experiment shows that when VEGFA expression is reduced, cell proliferation is reduced in TPC-1 as well as BCPAP cells; e, f. Transwell experiments shows that the cell migration capacity is reduced when VEGFA expression is reduced in BCPAP and TPC-1 cells; g, h. Transwell experiments shows that cell aggression is reduced when VEGFA expression is reduced in BCPAP and TPC-1 cells. (** P < 0.01)

In order to further explore the specific role of *VEGFA* in the development of PTC, we tested its influence on cell proliferation capacity via a CCK-8 test. The results suggested that the cell proliferation capacity was significantly inhibited Figure 6(a,b) after the reduction of *VEGFA* expression in the TPC-1 and BCPAP cells. The EdU experiment revealed the same outcome Figure 6(c,d). We used the transwell assay to detect cell migration and invasion and found that, after reducing *VEGFA* expression, the cell migration capacity was significantly reduced Figure 6(e,f) and the invasion capacity was also significantly inhibited Figure 6(g,h). Thus, we concluded that reduced *VEGFA* expression can significantly inhibit the proliferation, migration, and invasion of PTC cells.

### Downregulation of VEGFA inhibited the Wnt/β-catenin signaling pathway

**Figure 7.**
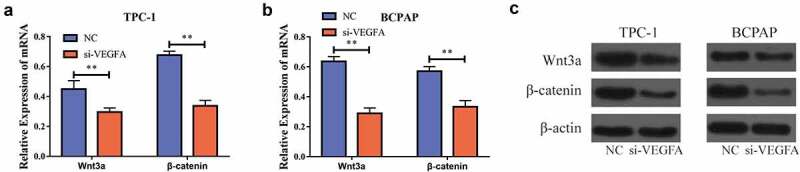
VEGFA could activate the Wnt/β-catenin signal pathway. a. Down-regulation of VEGFA expression in TPC-1 cells inhibits the mRNA expression of Wnt/β-catenin signal pathway-related genes; b. In BCPAP cells, when VEGFA expression is downregulated, the mRNA expression of Wnt/β-catenin signal pathway-related genes is inhibited; c. Western blot shows that when reducing VEGFA expression in BCPAP as well as TPC-1 cells, the protein levels of Wnt/β-catenin signal pathway-related genes decrease. (** P < 0.01)

In order to further elucidate the mechanism by which *VEGFA* is involved in the development of PTC, we tested the expression of Wnt/β-catenin signaling pathway-related genes through RT-PCR after lowering *VEGFA* expression in the cells. Upon lowering the *VEGFA* expression, the level of mRNA expression for the Wnt/β-catenin signaling pathway-related genes decreased significantly Figure 7(a,b), and the Western blot also showed significantly lower Wnt/β-catenin signaling pathway-related protein levels Figure 7(c). Taken together, we found that the Wnt/β-catenin signaling pathway-related gene expression also significantly inhibited *VEGFA* expression, and that *PVT1* activated the Wnt/β-catenin signaling pathway by targeting *miR-195* to upregulate *VEGFA* expression.

## Discussion

In recent decades, the incidence of TC has been increasing, and it is the most common incretory tumor [43,44]. PTC is the main type of TC, accounting for approximately 90% of cases and with an overall 5-year survival rate of approximately 97% [45,46]. However, the clinical prognosis of late PTC is poor, partly because the molecular mechanism of PTC occurrence and development is not adequately understood. Therefore, finding new biomarkers and therapeutic targets to improve the diagnosis and therapeutic outcomes of PTC is a clinical problem.

Many ncRNAs have been shown to regulate the expression of genes in cancer cells and are expected to become molecular biomarkers of cancer [12,47]. The structure of circRNAs has been shown to be highly stable and resistant to RNA degradation pathways. This suggests that circRNAs may be more technically suitable as molecular biomarkers for human cancer. In recent years, many studies have shown that circRNAs are involved in the inniniation and development of PTC. Luo et al. identify that *hsa_circ_0001018* can promote PTC by facilitating cell survival invasion, G/S cell cycle progression, and repress cell apoptosis via crosstalk with *miR-338-3p* and SRY-box transcription factor 4 (*SOX4*) [48]. Chu et al show that circular RNA *circRUNX1* can promote PTC progression and metastasis by sponging *miR-296-3p* and regulate DHD domain containing 2 (*DDHD2)* expression [49]. Ye and colleagues have demonstrated that circular RNA *circFOXM1* may play a vital role in PTC by sponging *miR-1179* and regulate high mobility group box 1(*HMGB1*) expression [50].

In this study, we found that *circPVT1* had significantly increased expression in PTC tissue. Specifically, *PVT1* expression level had a significant correlation with tumor size, TNM stage, and lymphatic metastasis in patients with PTC. In addition, we also found that the *circPVT1*expression level was significantly increased in the PTC cell lines compared to the Nthy-ori 3–1 cells. However, due to the limited number of specimens we collected and the short follow-up time, we have not yet observed the correlation between the expression level of CircPVT1 and the prognosis of PTC patients. At present, we have collected more specimens, and follow-up information will be improved. We will further verify the potential of circPVT1 as a diagnostic marker for PTC.

Then, we dectected the effect of *circPVT1* on cell proliferation, migration and invasion. After reducing the expression level of *circPVT1* in PTC cells, the ability of cell proliferation, migration, and invasion were significantly inhibited, and the *Wnt/β-catenin* signaling pathway was also inhibited. The *Wnt/β-catenin* signaling pathway, also known as the classic Wnt signaling pathway, has been studied in-depth [51,52]. Activation of the classic Wnt signaling pathway can contribute to extracellular *Wnt* combining with the transmembrane receptors *LRP5/6* and *Fzd* to form a complex. These receptors then become activated, which in turn activates the intracellular Disheveled protein, causing the inhibition of glycogen synthase enzyme-3 activity and subsequent detachment from the axin protein. This prevents formation of the *β-catenin* degradation complex, which in turn, prevents phosphorylation and degradation of *β-catenin*, thereby increasing the intracellular *β-catenin* expression. When *β-catenin* reaches a certain level, free *β-catenin* undergoes nuclear transfer and binds to the transcription factor *TCF/LEF* to form a transcription-activated complex. This complex eventually enhances or weakens the expression of certain genes. The *Wnt/β-catenin* signaling pathway has also been proved to be involved in the progression of PTC [53–55].

Previous studies documented that circRNAs were involved in regulating diverse biological processes via functioning as competing endogenous RNAs for miRNAs [37,38]. *CircPVT1* has been proved to locate mainly in cytoplasm, which meant that it might act as microRNA sponges to regulate downstream gene expression. *MiR-195* is dysregulated in several tumors, such as colon cancer [56], non-small lung cancer [57], laryngeal cancer [58,59], gastric cancer [60], cervical cancer [61], colorectal cancer [62], breast cancer [63], lung adenocarcinoma [64] and osteosarcoma [65]. Moreover, miR-195 has been demonstrated that play a significant role in PTC [66–69]. *VEGFA* is a powerful and specific endothelial cell fissure agent that, in combination with receptors, can participate in promoting the division and proliferation of vascular endothelial cells and increase microvascular permeability [70,71]. *VEGFA* has been shown to play an important role in tumor development. *VEGFA* is not only important to endothelial cell survival but also helps circulating endothelial cells reach new blood vessels. This in turn can promote tumor growth and angiogenesis, and thus *VEGFA* expression may become a new tumor biomarker [72,73]. *VEGFA* also participates in the development of PTC [74]. In this work, by using the dual luciferase reporter gene and Western blotting, we found that *circPVT1* expression can be increased by targeting *miR-195* to increase *VEGFA* expression. We have also shown in numerous experiments that the *Wnt/β-catenin* signaling pathway was inhibited and the cell proliferation, migration, and invasion abilities decreased after the reduction of *VEGFA* expression in cells. In summary, we found that *circPVT1* could activate the Wnt/β-catenin signaling pathway to promote the development of PTC.

Of course, this article still has many shortcomings. First of all, we have not conducted in vivo experiments to further verify the biological effects and molecular mechanisms of *circPVT1*. Secondly, there are multiple binding sites for miRNAs on *circPVT1*. It remains to be explored whether *circPVT1* binds to which miRNA plays the most important role. Thirdly, many studies have reported that ncRNAs have the potential to be developed as reagents for clinical treatment [75,76],whether *circPVT1* can be used as a therapeutic target for corresponding research and development still needs a lot of experimental proof. Moreover, we also need to collect more clinical specimens to explore the clinical application potential of *circPVT1*.

## Conclusion

Overall, our study demonstrated that *circPVT1* expression was frequently increased in PTC and high expression level of *circPVT1* was related to tumor growth and metastasis and poor prognosis. From the perspective of mechanism, *circPVT1* could function as a molecule sponge for *miR-195* and mediated the ceRNA network to promote the expression of *VEGFA*, finally promoted the malignant progression of PTC. Therefore, the *circPVT1*/ *miR-195*/*VEGFA* axis might be considered as a novel biomarker for prognosis and a promising therapeutic target for PTC treatment. Nonetheless, the current study only presents a theoretical basis for the mechanism of *circPVT1* involvement in PTC. Further research is required to explore the specific mechanisms involving various other molecular pathways involved in PTC carcinogenesis.

## Data Availability

The data used to support the findings of this study are available from the corresponding author upon reasonable request.
